# Health-Related Quality of Life and Survival of Cholangiocarcinoma Patients in Northeastern Region of Thailand

**DOI:** 10.1371/journal.pone.0163448

**Published:** 2016-09-29

**Authors:** Somkiattiyos Woradet, Nopparat Songserm, Supannee Promthet, Donald Maxwell Parkin

**Affiliations:** 1 Department of Public Health, Faculty of Health and Sports Science, Thaksin University, Phatthalung, Thailand; 2 Department of Community Health, Faculty of Public Health, Ubon Ratchathani Rajabhat University, Ubon Ratchathani, Thailand; 3 Department of Epidemiology, Faculty of Public Health, Khon Kaen University, Khon Kaen, Thailand; 4 ASEAN Cancer Epidemiology and Prevention Research Group, Khon Kaen University, Khon Kaen, Thailand; 5 Clinical Trial Service Unit & Epidemiological Studies Unit, University of Oxford, Oxford, United Kingdom; University Hospital Oldenburg, GERMANY

## Abstract

In northeast Thailand, cholangiocarcinoma (CCA) is a major cause of mortality. Patients with CCA have a poor prognosis and short-term survival. The purpose of this study was to investigate the association between health-related quality of life (HRQOL) and survival time, and to explore whether change in HRQOL score is related to survival among CCA patients. The study was performed between February 2011 and January 2012, and included 171 patients with newly diagnosed CCA from 5 tertiary hospitals in four provinces of northeast Thailand. The HRQOL was measured at baseline, 1 month, and 2 months after diagnosis by the FACT-Hep questionnaire (Thai version 4). The outcome was survival time from diagnosis. Cox’s proportional hazard model was used to evaluate the association between HRQOL and survival time. A higher overall score on HRQOL was associated with a significantly better survival (HR per 5 units increase in HRQOL was 0.92, 95% CI: 0.88–0.96). Two of the separate domains contributing to the overall HRQOL—functional well-being and hepatobiliary cancer subscale—were found to have independent effects on survival, even after adjustment for potential confounding variables, and the other domains of HRQOL. CCA patient whose HRQOL scores had improved (≥9 units) at the 1st month of follow up had a reduced probability of dying from the disease (HR: 0.56, 0.32–0.95) after adjustment for the same confounding factors. A positive association between HRQOL at diagnosis and survival time was found. An improvement in HRQOL score in the first months after diagnosis further increases survival.

## Introduction

Worldwide, cancer is responsible for 8.2 million deaths, or 22% of all deaths due to non-communicable diseases (NCD) [1], and the second most common cause of death from cancer in 2012 was cancer of the liver [2]. Almost 85% of the cases occur in low- and middle-income countries [2]. Cholangiocarcinoma (CCA) is the second-most common type of primary liver cancer and accounts for an estimated 15% of primary liver cancer worldwide [3]. However, the incidence of CCA in northeastern Thailand is very high (89.2 and 35.5 per 100,000 population in males and females, respectively [4]), due to endemic infection with the liver fluke *Opisthorchis viverrini*, and in this region it accounts for 60–90% of cases of primary liver cancer [4–6].

Normally, CCA patients have poor prognosis, with restricted treatment options. Most cases present with highly lethal tumors which are clinically silent in the early stage of disease, and are usually diagnosed at an advanced stage [7, 8]. Previous studies have shown the median survival of CCA patients to be about 4–5 months [8–10] and the six-month survival is only 35% [10]. Survival time is increased among CCA patients receiving surgery—an average of 36 months among patients with surgical treatment compared with 9 months in patients without surgery [11]. However, only about one in ten patients are eligible for some form of curative surgery [8].

“Quality of life” (QOL) is defined as the individual’s perception of their position in life within the context of the culture and value systems in which they live, and in relation to their goals, expectations, standards, and concerns [12]. Health-related quality of life (HRQOL) is an invisible outcome, but very crucial to the evaluation of the impact of treatment from the patient’s perspective. The measurement of HRQOL as an end point has become routine in clinical assessment, and is routinely included in studies of the efficacy of new treatments, including those for hepatobiliary cancers [13].

There is very little information about HRQOL among CCA patients, and its relationship to survival time, although four previous studies of the impact of HRQOL on survival time in hepatocellular carcinoma (HCC) and CCA have been reported. Two studies (one in China, one in France) demonstrated that QOL was positively associated with survival time among patients with advanced HCC [14, 15], while, in contrast, a study in China found that QOL did not predict survival in HCC patients [16]. However, these studies may not be very relevant to CCA patients, who have rather different clinical manifestations; in addition, these previous studies used a general test of QOL, rather than one adapted to the assessment of hepatobiliary cancer. One study in USA found that HRQOL was significantly associated survival of patients with HCC and CCA. The results showed that a hepatobiliary module of the FACT-Hep (symptoms and side- effects) was significantly associated with survival after adjusting for demographic, disease-specific, and treatment factors (middle tertile: HR = 1.98, 95% CI: 1.07–3.67 p-value = 0.028, highest tertile: HR = 1.97, 95% CI: 1.09–3.57 p = 0.025 compare with lowest tertile) [17]. However, this study showed result of overall patients which included HCC and CCA. The present study aims to estimate the association between HRQOL (overall, and of its different components) and survival in newly diagnosed CCA patients using a rigorous methodology, and an instrument specifically designed for the assessment of HRQOL in hepatobiliary cancer.

## Patients and Methods

### Study design

The subjects recruited into the study were all newly diagnosed as CCA by at least one of the following six diagnostic procedures: ultrasonography (U/S), computerised tomography (CT), magnetic resonance imaging (MRI), magnetic resonance cholangiopancreatography (MRCP), endoscopic retrograde cholangiopancreatography (ERCP), and histology. The subjects were recruited between February and July 2011 from the 5 tertiary hospitals serving four provinces of northeast Thailand (Srinagarind Hospital, Khon Kaen Regional Hospital, Maha-Sarakham Provincial Hospital, Kalasin Provincial Hospital, and Roi-Et Provincial Hospital) as described elsewhere [10]. A total of 237 patients with CCA were observed and followed-up in both hospital and community until the end of the study at 31^st^ January, 2012. The diagnosis date was the date that patients first presented themselves at those tertiary hospitals and were diagnosed by a physician as CCA.

### Ethical approved

This present study was approved by the Khon Kaen University Ethics Committee for Human Research, based on the Declaration of Helsinki and the ICH Good Clinical Practice Guidelines (Reference No. HE532325). Written informed consent was obtained from all patients.

### Independent variable

The independent variable in this study was the HRQOL score. It was collected using the Functional Assessment of Cancer Therapy-Hepatobiliary (FACT-Hep) Version 4 by Functional Assessment of Chronic Illness Therapy. More details and downloads are available from www.facit.org. Permission for the use of the HRQOL questionnaire was obtained for this study (letter of permission, dated December 29, 2010). This instrument is mostly widely used for patients with hepatobiliary cancers, including hepatocellular carcinoma (HCC), pancreatic cancer, gallbladder cancer, and CCA [18]. It is divided into two parts; the first relates to the general quality of life of cancer patients (Functional assessment of cancer therapy-general: FACT-G) and consists of 4 subscales (physical well-being, social/family well-being, emotional well-being, and functional well-being) with 27 items, and the second relates to the specific quality of life of CCA patients (hepatobiliary subscale) consisting of 18 items. The Thai version of the FACT-G was used [19–24]. Items of FACT-Hep are scored on a 5-point scale (0 = “not at all”, 4 = “very much”). The item on “satisfaction in sex life” was omitted because most patients preferred not to answer. The raw score on FACT-Hep, calculated using the guidelines, yielded a range of score from 0–176, with higher scores indicating a better HRQOL. All domains and overall FACT-Hep score variables were dichotomized using the median to yield “good” or “poor” scores. The information on the instrument used to assess HRQOL has been described in our previous publication [25].

The HRQOL score was collected by specially trained research assistants in each hospital at the time of enrollment into the study, and by the researcher in hospital or in the community among survivors at 1 and 2 months post-diagnosis. Monitoring and quality control procedures were established at the beginning of the study to ensure that there was maximum reliability and validity. For some of the confounders (e.g. conventional treatment at time of diagnosis or two months past diagnosis) were collected from the medical records.

### Outcome variables

The outcome was the survival time from CCA diagnosis until death or the end of study at 31^st^ January, 2012. Death status (cause of death and date of death) was confirmed by linkage with the death certificates from the Civil Registration system. Survival was confirmed by a telephone call to the patient or public health officer in community health centers. Censored data were defined as alive at the end of study, or death unrelated to CCA during the study period.

### Statistical methods

The Kaplan-Meier method was used to estimate the primary outcome of time from CCA diagnosis to death, and survival time was compared between subjects with poor and good scores for HRQOL.

The associations between survival and HRQOL scores were measured using Cox’s proportional hazard model, with results presented as hazard ratios (HR) and 95% confidence intervals (95% CI). Statistical analysis was performed with Stata version 12.1. As well as the HRQOL score, the models used included all the potential confounding variables found to be associated with HRQOL and/or survival in previous studies (sex, age at recruitment, stage at diagnosis, jaundice, ascites, carcinoembryonic antigen (CEA), conventional treatment, and use of alternative medicine) [10, 25]. The overall HRQOL score was included as a continuous variable, and the HR for a 5 unit change in overall HRQOL score was estimated. The contributions of the different domains of the baseline HRQOL to survival were analyzed by fitting each domain in a separate model as a binary variable, based on median-dichotomized scores.

The association between change in HRQOL score between baseline and 1 month, and 2 months post-diagnosis was analyzed using the fully adjusted Cox’s proportional hazard model. A change of 8 units of HRQOL score was considered clinically meaningful [13], so change was categorized to 4 groups (no change (-8 to 8 units change), decrease (≤ -9 unit’s change), improvement (≥ 9 units’ change), and an unknown group (most of this group died before the 1- or 2-month follow up, with a very few lost to follow up)).

For missing data; those still alive were all available for the 1- and 2 month assessments post diagnosis. None of the subjects refused to respond to the FACT-HEP at baseline or at the 1- and 2-month assessments and the research assistants and the researcher always ensured that all subjects provided responses to the items. For any missing data occurred we assumed to be missing at random (i.e. the missing data will not bias the outcome), then it can be ignored, and the analyses based solely on the available data.

## Results

237 patients with suspected CCA were recruited and interviewed. 66 out of 237 cases (27.8%) were excluded because they did not meet the criteria for a confirmed diagnosis by 1 of 6 diagnostic procedures. 171 CCA patients were available for analysis contributing a total of 758.4 person-months of follow-up. Of these, 128 died during the interval—a mortality rate of 16.9 per 100 person-months (95% CI: 14.1–20.1). There were 99 cases that completed follow-up for HRQOL over time. The six-month survival was 35.7%, and median survival time 4.3 months (95% CI: 3.3–5.1) [10].

The subjects were aged between 37 and 86 (mean 63.6), about two-thirds (64.9%) were male and 43.3% were aged 60–69 years. Most patients were married (80.7%), of low educational level (91.2% primary school or less) and four-fifths were farmers or agricultural labourers. Two-thirds of patients (64.3%) had household income less than or equal 5,000 Baht per month (US$165 in 2011) and average household income was 6,936 (SD: 8,074) Baht per month ($230). 49 (28.7%) of patients had been diagnosed by CT or MRI or MRCP and tumour marker, 92 (53.8%) were classified as being at an advanced stage, 53.2% presented with jaundice and 17.5% with ascites, and 66 (38.6%) were positive for CEA. About one-third of the patients (33.9%) received standard treatment which included chemotherapy, surgery and radiation, and 77 (45.0%) had used alternative medicine as described elsewhere [10].

The domain of HRQOL with the highest score at baseline was social/family well-being (average score: 3.66) and the lowest was in the functional well-being domain (2.72) (Table 1).

**Table 1:** Base line HRQOL score of CCA patients assessed by FACT-Hep.

Variable	N	Median	Mean	S.D.	Average score (0–4)
Physical well-being (0–28)	171	18	18.25	5.14	2.61
Social/family well-being (0–24)	171	24	21.93	2.93	3.66
Emotional well-being (0–24)	171	17	16.92	4.70	2.82
Functional well-being (0–28)	171	18	19.04	6.77	2.72
Hepatobiliary cancer subscale (0–72)	171	49	49.39	11.03	2.74
Total (overall HRQOL) (0–176)	171	125	125.54	23.97	2.85

Multivariate analysis using Cox’s proportional hazard model was performed to investigate the association between HRQOL and survival time from diagnosis to death. Overall HRQOL score was related to survival, with a 5 point increase in score being associated with a statistically significant (P<0.001) decrease of 8% in survival, and this result remained unchanged (HR: 0.92, 05% CI: 0.88–0.96) even after adjustment for those confounding factors which had been found to be associated with HRQOL and survival (sex, age, stage of disease, present of jaundice and ascites, a positive CEA, and treatment (conventional and alternative)).

**Fig 1:**
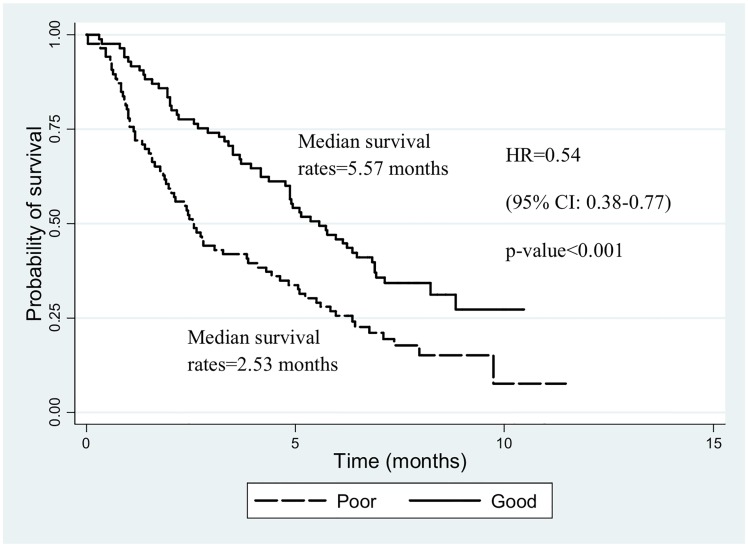
Kaplan-Meier survival estimate of time from diagnosis to time of death by HRQOL score in the CCA patients assessed by FACT-Hep.

Fig 1 shows the Kaplan-Meier survival curves for patients with overall HRQOL scores above or below the median score of 125. The median survival rates of patients with above HRQOL scores was 5.57 month and below HRQOL scores was 2.53 month (HR = 0.54: 95% CI: 0.38–0.77, p-value<0.001).

Table 2 shows the contribution of the different domains of the HRQOL to survival. The HR was significantly lower among subjects with scores above the median value on the physical well-being, functional well-being, and hepatobiliary cancer subscales, even after adjustment for the confounding variables described earlier. However, after including the score on the hepatobiliary cancer subscale in the model (along with the 8 potential confounders) the association between survival and a good score on the physical well-being subscale was no longer statistically significant (0.69; 0.42–1.12), while the association with a high score for functional well-being was reduced (0.61; 0.41–0.91), but remained significant (P = 0.016).

**Table 2:** Crude and adjusted analysis of association between scores on the component domains of the HRQOL scale assessed by FACT-Hep and mortality of CCA patients.

Domains^a^	N	Median Time	Crude HR	Adjust HR^b^	95% CI	p-value
Physical well-being						0.002
• poor (<18)	81	2.40	1.00	1.00		
• good (≥18)	90	5.57	0.51	0.53	0.35–0.79	
Social/family well-being						0.212
• poor (<24)	77	3.17	1.00	1.00		
• good (≥24)	94	4.77	0.94	0.78	0.53–1.15	
Emotional well-being						0.209
• poor (<17)	84	4.10	1.00	1.00		
• good (≥17)	87	4.30	1.04	0.79	0.54–1.14	
Functional well-being						0.002
• poor (<18)	80	2.57	1.00	1.00		
• good (≥18)	91	5.50	0.59	0.55	0.37–0.81	
Hepatobiliary cancer subscale						<0.001
• poor (<49)	78	2.37	1.00	1.00		
• good (≥49)	93	5.77	0.45	0.51	0.35–0.76	
Physical well-being						0.135
• poor (<18)	81	2.40	1.00	1.00		
• good (≥18)	90	5.57	0.51	0.69^c^	0.42–1.12	
Functional well-being						0.016
• poor (<18)	80	2.57	1.00	1.00		
• good (≥18)	91	5.50	0.59	0.61^c^	0.41–0.91	

^a^Each domain is an individual multivariate model.

^b^Adjusted for 7 potential confounders: sex, age, stage, ascites, carcinoembryonic antigen, conventional treatment, and use of alternative medicine.

^c^Adjusted for 7 potential confounders and hepatobiliary cancer subscale.

The association between survival and the change in HRQOL score between baseline and 1 month, and between baseline and 2 months, was as shown in Table 3. Patients showing an improvement in HRQOL at 1 month had a statistically significant reduced risk of death compared with those who had no change in HRQOL (0.56; 0.32–0.95, after adjustment for the confounding variables). Only 99 subjects survived to 2 months, so that the lower HR in those showing an improved HRQOL score (0.68; 0.35–1.31) was no longer statistically significant.

**Table 3:** Crude and adjusted analysis of association between change in HRQOL score assessed by FACT-Hep at 1 and 2 month and survival time from CCA diagnosis to death.

Factors	N	Crude HR	Adjusted HR^a^	95% CI	p-value^b^
Change in HRQOL score at 1 month					<0.001
• No change (-8 to 8 units)	32	1.00	1.00		
• Decrease (≤-9 units)	32	1.47	1.10	0.61–1.97	
• Improve (≥ 9 units)	67	0.79	0.56	0.32–0.95	
• Unknown	40	5.00	4.23	1.68–10.65	
Change in HRQOL score at 2 month					<0.001
• No change (-8 to 8 units)	22	1.00	1.00		
• Decrease (≤-9 units)	26	1.24	1.28	0.64–2.56	
• Improve (≥ 9 units)	51	0.54	0.68	0.35–1.31	
• Unknown	72	7.18	7.44	3.67–15.10	

^a^adjusted for potential confounders: sex, age, stage, jaundice, ascites, carcinoembryonic antigen, conventional treatment, and use of alternative medicine.

^b^p-value from partial likelihood ratio test.

## Discussion

Our study shows that HRQOL, measured at the time of diagnosis, predicts survival probability in patients with CCA in North Eastern Thailand, and that some of the components making up the HRQOL scale—notably those including specific aspects of hepatobiliary cancers, or with physical or functional well-being are the most relevant in this respect.

The advantage of the present study is the relatively large sample size, the unselected nature of the patients studied, prospective design, and the rigorous statistical methodology used to quantify the associated between HRQOL and mortality of the subjects. In addition, this study used a HRQOL questionnaire specific for hepatobiliary cancer (FACT-Hep), which is more appropriate to assess HRQOL in CCA patients. All factors that had been shown in previous studies [10, 25] to be significantly associated with HRQOL were considered as potential confounding factors and included in the multivariable model.

A limitation of the study is that relatively few of the patients had been diagnosed as CCA by histology, so it is possible that other types of cancer had been misclassified as CCA. Although most CCA patients were not histologically diagnosed, all were diagnosed by clinicians in tertiary hospitals in the northeast region of Thailand where there is a high incidence of CCA, and considerable local expertise and experience in diagnosis of CCA is available.

Our observation that patients who had higher HRQOL score had lower fatality is consistent with review of Montazeri [26] which found that most studies show a significant independent predictor of survival duration among various cancer patients.

The components of the HRQOL most relevant to survival were physical well-being, functional well-being, and hepatobiliary cancer subscale. The latter, in particular, includes dimensions related to symptoms and clinical manifestation of CCA disease (and related physical and biological factors) that might affect to survival among CCA patients. Previous studies [14, 16, 17, 27–29] have also shown that physical and functional (or role) domains of HRQOL were significantly associated with survival time in cancer patients. The social/family and emotional well-being subscales did not show any association with survival time of suspected CCA patients in our study. Previous prospective studies (involving various types of cancer) have found a similar lack of association [16, 17, 28, 30] or the converse- that social and emotional domains were associated with survival [27–29].

We found that an improving HRQOL score post diagnosis was particularly associated with a statistically significant reduced risk of mortality. A possible explanation is that newly diagnosed CCA patients have a degree of uncertainty about their immediate future, and this has a direct effect on HRQOL [31], while after early treatment they feel more confident. Alternatively, a relatively favourable clinical response to treatment (with consequently improved prognosis) is likely to engender an increase in the HRQOL score.

Our study, like previous ones using patients with other forms of cancer, has shown that a higher HRQOL score is associated with a better survival. The reasons for this link still debated. It is possible that HRQOL is related to some biological or clinical factors of prognostic importance that are not measured, or adjusted for in the analysis. On the other hand, it is possible that HRQOL is a marker of characteristics such as personality style, or adapting/coping strategies, which have been postulated to affect disease processes, adherence to treatment, and outcome among cancer patients [32]. A causal association can really only be inferred through intervention studies, but, although some such studies have been performed [33], the possibility that psychosocial intervention can have an effect on survival remains contentious [34]. Perhaps the more immediate relevance is in clinical decisions on treatment, when prolongation of life can be achieved at the cost of loss of quality; patient involvement in decision making is feasible and desirable [35].

## Supporting Information

S1 Dataset _SW.xlsx. HRQOL and Survival of CCA Patients in Northeast Thailand.
(XLSX)
